# Effects of Qigong Training on Health-Related Quality of Life, Functioning, and Cancer-Related Symptoms in Survivors of Nasopharyngeal Cancer: A Pilot Study

**DOI:** 10.1155/2014/495274

**Published:** 2014-05-28

**Authors:** Shirley S. M. Fong, Shamay S. M. Ng, W. S. Luk, Louisa M. Y. Chung, Janet Y. H. Wong, Joanne W. Y. Chung

**Affiliations:** ^1^Institute of Human Performance, University of Hong Kong, Pokfulam, Hong Kong; ^2^Department of Rehabilitation Sciences, Hong Kong Polytechnic University, Hung Hom, Hong Kong; ^3^The Association of Licentiates of the Medical Council of Hong Kong, Hong Kong; ^4^Department of Health and Physical Education, Hong Kong Institute of Education, Tai Po, Hong Kong; ^5^School of Nursing, Li Ka Shing Faculty of Medicine, University of Hong Kong, Pokfulam, Hong Kong

## Abstract

This study aimed to investigate the effects of Qigong intervention on quality of life (QOL), health-related functioning, and cancer-related symptoms in survivors of nasopharyngeal cancer (NPC). Twenty-five survivors of NPC were included in the experimental group (mean age ± SD: 55.4 ± 7.5 years) and 27 in the control group (mean age ± SD: 58.7 ± 9.5 years). The experimental group underwent a weekly 1.5-hour Qigong training program and an identical home program (three times/week) for six months. The control group received no training. Global health status/QOL, functioning, and cancer-related symptoms were assessed by the European Organization for Research and Treatment of Cancer QLQ-C30 and QLQ-H&N35 questionnaires before training began, after three months of Qigong training, at the end of the six-month Qigong intervention (i.e., posttest), and six months posttest. Intention-to-treat analysis revealed no statistically (*P* > 0.05) or clinically significant improvement in global health status/QOL, functioning, or symptoms in either group. The experimental group had 45.8% fewer sense-related (smell and taste) problems (*P* < 0.05) but 98.6% more speech-related problems (*P* < 0.05) than the control group after the Qigong intervention. Qigong training resulted in no apparent improvement in health-related QOL, functionality, or cancer-related symptoms in cancer-free survivors of NPC, except for a possible reduction in smell- and taste-related problems.

## 1. Introduction

Many people in Southeast Asia, Southern China, and North Africa are diagnosed with nasopharyngeal cancer (NPC), a disease endemic in these regions [1]. The incidence rate is high in these areas, ranging from 25 to 50 per 100,000 persons [1]. Although advances in NPC treatments have led to improvements in survival rates (five-year survival rate = 55%–90%) [2], survivors often live with numerous cancer- or treatment-related symptoms that cause dysfunctions and a reduction in quality of life (QOL) [3, 4]. A previous study reported that survivors of NPC had a lower health-related QOL during their cancer-free survival period than the healthy control participants. Socioeconomic status was the major determinant of QOL in this group. Participants who had a lower economic status tended to have a lower QOL [3]. It is thus necessary to identify low-cost remedial strategies to improve QOL and reduce adverse side effects in survivors of NPC.

Qigong is a low-cost mind-and-body exercise that originated in China. It comprises meditation, coordinated breathing, and graceful bodily movements [5]. There is some evidence that Qigong can improve QOL and reduce side effects of cancer treatment [6–8]. Several studies have reported the positive effects of Qigong training on global QOL as measured by the European Organization for Research and Treatment of Cancer (EORTC) Quality of Life Core Questionnaire 30 (QLQ-C30) [7, 8]. Cancer treatment side effects, such as constipation [7] and inflammation, were reduced [7, 8] and perceived cognitive abilities improved [8]. These promising results reported in cancer patients raised the possibility that survivors of NPC may also benefit from Qigong training after the conventional treatment period. To date, no study has examined the effect of Qigong intervention on residual cancer- or treatment-related side effects and QOL in NPC survivors, who tend to be young and active members of the workforce [2].

The aim of this prospective, nonrandomized, and controlled trial was to investigate the effects of Qigong training on health-related QOL, functions, and cancer-related symptoms in survivors of NPC. The research hypotheses of this study were (1) improvements in QOL and functioning and a reduction in symptoms should be observed in the experimental group across the four assessment time points (i.e., pretest, midintervention, posttest, and follow-up) and (2) the experimental group should have better QOL, higher functions, and fewer cancer-related symptoms than the control group immediately after Qigong intervention and also at the six-month follow-up point.

## 2. Methods

### 2.1. Participants

Survivors of NPC were recruited from a medical clinic and a cancer self-help group by convenience sampling. They were first screened by a medical doctor to ensure that inclusion and exclusion criteria were met. The inclusion criteria were as follows: (1) individuals had a history of NPC (i.e., positive Epstein-Barr virus DNA and biopsy test results) but were free from the disease during the study period; (2) had completed all cancer treatments (e.g., chemotherapy and radiotherapy) although conventional medical care was allowed; (3) were medically stable; (4) aged between 40 and 85 years; (5) had expected survival period of more than one year; (6) spoke Cantonese; (7) residing in Hong Kong; (8) had normal cognitive function; and (9) could read Chinese. The exclusion criteria were (1) receiving alternative and complementary therapies (e.g., traditional Chinese medicine); (2) having significant chronic medical conditions such as uncontrolled hypertension; (3) having significant neurological, musculoskeletal, cardiopulmonary, or vascular disorders; (4) ambulating dependently; and (5) smoking. Ethical approval was obtained from the ethics review committee of the administering institutes. Written informed consent was obtained from each participant prior to data collection. All procedures were conducted according to the Declaration of Helsinki principles.

### 2.2. Intervention

The intervention group received weekly 1.5-hour face-to-face Qigong training continuously for six months. The traditional 18 Forms Tai Chi Internal Qigong [5] and NPC-targeted exercises were included in the Qigong training program (Table 1). This training protocol specific for survivors of NPC was designed by a medical doctor and a Qigong master. It places more emphasis on the olfactory, inner ear, and oral stimulations when compared to the traditional Qigong forms. Similar to other Qigong training regimes, our training program emphasized meditation, coordinated breathing, and slow and smooth Tai Chi movements. Participants progressively learned one to two new techniques in each face-to-face session (Table 1). A certified Qigong instructor and an assistant instructor delivered all the Qigong classes. Moreover, a Qigong home program was prescribed to the intervention group to increase the training frequency to four times per week in total. The home exercises were exactly the same as those learned in the supervised sessions. The Qigong instructors checked the participants' compliance to the home program by asking and testing them every week to ensure that the training volume was adequate. All participants stopped practicing Qigong during the follow-up period. The control group received no Tai Chi or Qigong training but continued to receive conventional medical care if necessary throughout the whole study period.

### 2.3. Outcome Measurements

Demographic characteristics, habitual physical activity level (metabolic equivalent hours per week), and medical history of the participants were obtained at pretest (Table 2). In addition, all of the participants were required to complete two questionnaires—EORTC QLQ-C30 and the head and neck cancer supplementary module (QLQ-H&N35)—to evaluate their global health status/QOL, functioning and cancer-related symptoms one week before the Qigong intervention (pretest), after three months of Qigong training (midintervention test), immediately after the six-month Qigong intervention (posttest), and six months posttest (follow-up test).

The EORTC QLQ-C30 (version 3.0 for the Hong Kong Chinese population) and the QLQ-H&N35 (Hong Kong Chinese version) were used together as originally designed [10]. These self-administered questionnaires were validated for the Hong Kong Chinese population and their psychometric properties are considered to be satisfactory [11, 12]. The QLQ-C30 questionnaire, suitable for a board range of cancer patients and survivors, comprises 30 items assessing health-related QOL. It is composed of one global health status/QOL scale, five functional scales (i.e., physical, role, cognitive, emotional, and social), three symptom scales (i.e., fatigue, nausea and vomiting, and pain), and six single items (i.e., dyspnea, insomnia, appetite loss, constipation, diarrhea, and financial difficulties). Items on the functional and symptom scales were scored with a four-point Likert scale (1 = not at all, 2 = a little, 3 = quite a bit, and 4 = very much) while items in the global health status/QOL domain were scored on a seven-point Likert scale, ranging from 1 (very poor) to 7 (very good) [10].

The QLQ-H&N35 module was used to assess symptoms specifically in head and neck cancer patients and survivors. It consists of 35 items in total that were scored with a four-point Likert scale (1 = not at all, 2 = a little, 3 = quite a bit, and 4 = very much). Seven scales assess symptoms of pain, swallowing, senses (smell/taste), speech, social eating, social contact, and sexuality, and six single items assess the presence of symptomatic problems associated with teeth, mouth opening, dry mouth (xerostomia), sticky saliva, coughing, and ill feeling [10]. Both the EORTC QLQ-C30 and QLQ-H&N35 were scored according to the EORTC QLQ-C30 scoring manual [10]. In brief, all of the scale and item raw scores were linearly transformed into a 0 to 100 scale. A higher score represents a higher level of functioning on the QLQ-C30 functional scale or a higher level of global health status on the global health status/QOL scale. However, a higher score on the QLQ-C30/QLQ-H&N35 symptom scale indicates more symptoms or problems, suggesting a greater impairment in QOL [10].

### 2.4. Statistical Analysis

The Kolmogorov-Smirnov test was used to check normality of all continuous data. Independent* t*-tests (for continuous variables) and chi-square tests (for categorical variables) were used to compare the demographic characteristics and all outcome variables of the Qigong and control groups before the training intervention began (baseline). Intention-to-treat analysis, specifically the last observation carried forward method, was employed to handle the missing data. To assess the time-by-group interaction effect of Qigong training on the global health status, two-way repeated measures analysis of covariance (ANCOVA) with mixed design (within-subject factor: time and between-subject factor: group) was used.

In addition, two-way repeated measures multivariate analysis of covariance (MANCOVA) was performed to evaluate the overall time-by-group interaction, group and time effects of Qigong training on (1) functional scale outcomes and (2) symptom scale outcomes of EORTC QLQ-C30 and QLQ-H&N35. Multivariate tests were used instead of univariate tests to avoid an increase in type I error associated with multiple comparisons. If there were significant between-group differences in any outcome variables at baseline, these outstanding outcomes were treated as covariates in the multivariate and univariate tests mentioned above. Effect sizes (partial eta-squared) were also reported. By convention, values of 0.14, 0.06, and 0.01 represent large, medium, and small effects, respectively [13].

If the two-way repeated measures MANCOVA or ANCOVA revealed significant effects overall, follow-up analyses were performed using one-way repeated measures analysis of variance (ANOVA) and post hoc paired* t*-tests with Bonferroni correction as appropriate. Independent* t*-tests were also used to compare the outstanding outcome variables between the two groups. The significance level (alpha) was set at 0.05 (two-tailed) for all the tests except the paired* t*-tests (alpha was set at 0.008 due to six pairwise comparisons). All statistical analyses were performed using the IBM Statistical Package for the Social Sciences (SPSS) version 20.0 software (IBM, Armonk, NY, USA).

## 3. Results

Fifty-two survivors of NPC were eligible to join the study and completed the baseline assessments. Twenty-five participants underwent Qigong training voluntarily for six months while twenty-seven participants acted as controls. At the end of the study, 14 participants in the experimental group had completed the Qigong training and the follow-up tests (i.e., dropout rate = 44.0%) and 21 participants in the control group had completed the follow-up tests (i.e., dropout rate = 22.2%). The reasons for dropping out were similar between the two groups. For the Qigong group, subjects who left the trial reported they were busy (*n* = 4), sick (*n* = 2), or unable to commit to the time (*n* = 5). Those control group subjects who stopped participating were busy (*n* = 1), unable to commit to the time (*n* = 1), overseas (*n* = 1), or unreachable (*n* = 3). No adverse effects were reported during the in-class Qigong training or the home practice. The Qigong class attendance rate was on average approximately 90%.

### 3.1. Comparison of Baseline Data

No significant differences were found in any of the demographic (Table 2) and outcome variables (Table 3) between the two groups at baseline (*P* > 0.05). Therefore, analysis of variance (ANOVA) instead of ANCOVA was performed in all the univariate and multivariate tests.

### 3.2. Global Health Status/QOL

Two-way repeated measures ANOVA results revealed that the group × time interaction and group effects were not significant (*P* > 0.05). However, the time effect was significant (*P* = 0.011) (Table 3). Post hoc paired *t*-tests showed that the global health status score, representing health-related QOL, was 12.6% higher at posttest (*P* = 0.001) and 15.9% higher at followup (*P* = 0.006) when compared to the pretest value of the control group. No significant differences (*P* > 0.05) were found in other pairwise comparisons between time points (Table 3).

### 3.3. EORTC QLQ-C30 Functional Scale Outcomes

The *P* values and effect sizes of the multivariate analysis are presented in Table 3. No significant group × time, group or time effects (*P* > 0.05) were found. Therefore follow-up univariate analyses were not performed.

### 3.4. EORTC QLQ-C30 Symptom Scale Outcomes

Similar to the QLQ-C30 functional scale outcomes, no significant group × time, group or time effects (*P* > 0.05) were found in the multivariate analysis (Table 3).

### 3.5. EORTC QLQ-H&N35 Symptom Scale Outcomes

MANOVA results showed that the group × time interaction and time effects were not significant (*P* > 0.05). Only the group effect was significant (*P* = 0.003). Follow-up independent* t*-tests identified significant between-group differences at (1) midintervention (*P* = 0.027), posttest (*P* = 0.017), and follow-up test (*P* = 0.006) for the sense problems outcome and (2) midintervention (*P* = 0.038) and posttest (*P* = 0.034) for the speech problems outcome (Table 3). The control group had higher scores for sense-related (smell and taste) problems but lower speech-related problem scores than the Qigong group during and after the Qigong intervention (Table 3).

## 4. Discussion

This is the first study to assess the impact of Qigong training on QOL for cancer-free survivors of NPC. Our results show that Qigong training did not improve health-related QOL for the intervention group. Instead global health status/QOL improved in the no-training control group. Our findings are in contrast to previous studies and do not support our hypotheses. Qigong has been proposed to improve QOL in cancer patients [7, 8, 14] through several possible mechanisms, including reducing the side effects of conventional cancer treatments and cancer-related symptoms [7], decreasing inflammation [7, 8], and increasing cognitive function [8]. A major reason for the discrepancy in findings between our study and the previous studies could be the difference in the participants' characteristics. Previous randomized controlled trials included heterogeneous cancer patients (e.g., breast cancer, lymphoma, and lung cancer) who had received/were receiving different forms of conventional cancer treatments. All of them were recruited from hospitals [7, 8]. Newly diagnosed cancer patients and patients in the treatment stage could be more prone to anxiety, depression, and other psychological impairments than rehabilitated survivors of cancer [15]. Hence, they may benefit more from Qigong training in terms of improving QOL [7, 8].

Our study included survivors of NPC exclusively. They were recruited from a medical clinic and a cancer self-help group. All of them were cancer-free during the study period. The post-NPC duration for the Qigong group was 12.5 years while for the control group it was 8.4 years. They may thus have fewer symptoms and functional impairments and so better health-related QOL overall when compared to hospitalized cancer patients. The mean global health status/QOL score of the Qigong group (61.8) actually exceeded the EORTC QLQ-C30 reference value (61.3) at pretest [16]. Thus, we postulate that Qigong training may not improve their relatively high level of QOL further. Instead, spontaneous improvement could heighten their health-related QOL, as demonstrated in the no-training control group. Further study should include a larger sample and adopt a randomized controlled study design to investigate this hypothesis. In addition, qualitative studies (e.g., structured interviews) could be carried out to discover the reasons for the spontaneous improvement in QOL among survivors of NPC.

The results also revealed that, over the study period, neither the Qigong group nor the control group showed significant improvements in physical, role, emotional, cognitive, or social functions. These findings are not entirely surprising given the fact that our participants had generally higher EORTC QLQ-C30 functional scores than the norm [16] at baseline. Perhaps no further improvements in functioning were possible regardless of the stimulus. This is the first study to include a group of rehabilitated survivors of NPC. Comparing our results with those obtained for cancer patients [7, 8] may not be appropriate. Further randomized controlled trials should be carried out to explore whether or not Qigong training can further improve functions in survivors of NPC.

Among all of the general cancer symptoms (QLQ-C30 symptom scores) and NPC-specific symptoms (QLQ-H&N35 symptom scores) measured, only the sense-related problems score was lower in the Qigong group than in the control group during and after the intervention period. The Qigong group continued to have fewer sense-related (smell and taste) problems than the control group up to six months after the cessation of training. Our findings are in line with a previous case report that suggested that smelling and taste in the mouth changed during Qigong practice [17]. These findings are encouraging because chronic smell and taste disorders are common among survivors of NPC [18, 19]. These consequences of NPC and/or side effects of chemotherapy and radiotherapy can impair food intake and so may lead to nutritional deficits and diminished QOL [18, 20, 21]. However, no effective curative strategy has been developed [18, 22, 23].

Qigong has potential use as a remedial intervention to improve perceived taste and smell in survivors of NPC. During the NPC-targeted exercises of the Qigong intervention, participants were requested to massage their oral cavity with their tongue (Table 1). We postulated that this technique might stimulate the taste buds, cortical representation of taste [24], and saliva production, which are essential for the taste sensation [18]. In addition, the olfactory stimulation exercises required the participants to breathe in slowly and deeply as if they were smelling a flower (Table 1). This kind of exercise might stimulate the olfactory receptors located in the nasal cavity, which are crucial for smell and taste sensations [18]. Further study should evaluate smell and taste sensations objectively, for example, using the “Sniffin' Sticks” test [19], to exclude possible psychological or placebo effects before suggesting Qigong training for NPC survivors.

Another statistically significant finding is that the Qigong group experienced more speech problems than the control group at posttest and follow-up test. This finding was unanticipated as a previous study found that Qigong could improve speech fluency in people who stutter, as they gained more control over muscular tension after the exercise [25]. The speech-related problems considered in the QLQ-H&N35 questionnaire concerned hoarseness and trouble talking to others [10], which may not be directly related to muscular control of sound production. Further study should explore whether the increased speech problems observed are due to Qigong training, aging, smell and taste problems, and so forth.

The Qigong training intervention was not able to alleviate symptoms such as fatigue, pain, insomnia, and swallowing problems in survivors of NPC (Table 3). This may be because our participants had been fully rehabilitated and did not have residual cancer-related problems [16]. As a result, Qigong training might not be able to further lessen their symptoms.

This study has several limitations and findings should be interpreted with caution. First, the group assignment was non-randomized. Participants in the Qigong group may have been more motivated to seek treatments or training, and, therefore, they may have had fewer symptoms (e.g., sense-related problems) than the control group [13]. Second, due to the nature of the Qigong intervention, blinding of participants was not possible. In addition, we used self-report questionnaires to measure outcomes, so bias/placebo effects (e.g., participants perceive they feel better after Qigong training) cannot be excluded. Third, the sample size is rather small that may have compromised the statistical power leading to the insignificant results [26]. Finally, all of the participants were cancer-free survivors. The results cannot be generalized to patients of NPC who may have more symptoms and poorer functions. The effect of Qigong training on patients with NPC requires further investigation. Moreover, further study should take confounding factors (e.g., social support) into account when measuring QOL in survivors of NPC.

## 5. Conclusions

In summary, this clinical trial indicated that Qigong training confers no apparent improvement on health-related quality of life, functioning, or cancer-related symptoms in survivors of NPC. Further study is required to determine whether the improvement in taste- and smell-related problems perceived by survivors of NPC during and after Qigong training was due to actual recovery of senses or due to the placebo effect only.

## Figures and Tables

**Table 1:** Qigong exercise protocol.

Exercise type	Duration
Warm-up: jogging	5 minutes
18 Forms Tai Chi Internal Qigong [5]	40 minutes
(1) Raising the arms and pressing down	
(2) Opening the chest	
(3) Painting a rainbow from side to side	
(4) Separating the clouds with two arms	
(5) Rolling the arms in a horse-riding stance	
(6) Rowing the boat	
(7) Carrying a ball in front of the shoulders	
(8) Turning around to look at the moon	
(9) Twisting the waist and pushing the palms	
(10) Cloud hands in a horse-riding stance	
(11) Scooping the sea and searching the sky	
(12) Pushing waves in walking stance	
(13) Flying dove spreads its wings	
(14) Punching in horse stance	
(15) Flying like wild geese	
(16) Rotating a flying wheel	
(17) Stepping whilst bouncing a ball	
(18) Pressing down to balance the chi	
Additional NPC-targeted exercises	40 minutes
(1) Arms cross in front and curl up the body	
(2) Place hands behind the neck	
(3) Olfactory stimulation (e.g., breathe in slowly and deeply, imagine smelling a flower)	
(4) Inner ear stimulation (e.g., different head and neck movements)	
(5) Oral and swallowing exercise (e.g., use the tip of the tongue to massage the maxilla and swallow saliva bit by bit)	
(6) Mouth-opening exercise and make sounds with the throat	
Cooldown: gentle whole-body stretching	5 minutes

**Table 2:** Characteristics of participants measured at baseline.

	Qigong group (*n* = 25)	Control group (*n* = 27)	*P*
Age (year)	55.4 ± 7.5	58.7 ± 9.5	0.172
Sex (number of male : female)	12 : 13	16 : 11	0.416
Weight (kg)	59.5 ± 15.1	56.8 ± 10.5	0.451
Height (cm)	163.2 ± 9.1	161.5 ± 8.1	0.496
Body mass index (kg/m^2^)	22.3 ± 5.0	21.7 ± 3.3	0.640
Reported NPC stage at diagnosis [9]			
Stage I (*n*, %)	5 (20%)	2 (7.4%)	
Stage II (*n*, %)	5 (20%)	7 (25.9%)	
Stage III (*n*, %)	11 (44%)	15 (55.6%)	
Stage IV (*n*, %)	4 (16%)	3 (11.1%)	
Post-NPC duration (year)	12.5 ± 7.1	8.4 ± 9.7	0.094
Previous NPC treatment			
Radiotherapy (*n*, %)	17 (68%)	9 (33.3%)	
Radiotherapy and chemotherapy (*n*, %)	7 (28%)	18 (66.6%)	
Radiotherapy, chemotherapy, and surgery (*n*, %)	1 (4%)	0 (0%)	
Habitual physical activity level (metabolic equivalent hours per week)	13.9 ± 14.1	14.9 ± 14.5	0.795

Mean ± standard deviation presented for continuous variables.

*P < 0.05 for between-group comparisons.

**Table 3:** EORTC scores measured at four time points.

	Qigong group (*n* = 25)				Control group (*n* = 27)				Group × time interaction		Group effect		Time effect	
	Pre	Mid	Post	FU	Pre	Mid	Post	FU	Effect size	*P*	Effect size	*P*	Effect size	*P*
EORTC QLQ-C30														
Global health status/QOL	61.81 ± 22.10	63.19 ± 24.32	61.81 ± 26.57	66.67 ± 27.47	58.65 ± 21.01	62.50 ± 18.30	66.03 ± 18.55^a^	67.95 ± 16.45^a^	0.143	0.067	<0.001	0.945	0.213	0.011*
Functional scales (higher scores = higher functions)									0.387	0.189	0.143	0.221	0.410	0.134
Physical functioning	78.06 ± 16.85	80.56 ± 15.22	79.17 ± 13.94	78.89 ± 15.06	76.67 ± 13.10	78.72 ± 12.22	77.18 ± 12.53	77.44 ± 13.21
Role functioning	80.56 ± 22.88	81.25 ± 21.03	84.03 ± 21.69	86.11 ± 21.80	87.18 ± 19.04	86.54 ± 18.27	85.90 ± 18.07	84.62 ± 21.56
Emotional functioning	76.74 ± 19.19	79.51 ± 19.35	78.13 ± 20.24	81.60 ± 18.39	77.24 ± 20.35	76.28 ± 18.96	78.53 ± 19.32	80.45 ± 18.40
Cognitive functioning	65.97 ± 23.30	65.28 ± 20.21	68.75 ± 24.23	69.44 ± 21.23	69.23 ± 24.81	67.95 ± 26.63	72.44 ± 22.58	69.87 ± 24.50
Social functioning	74.31 ± 25.53	75.69 ± 26.00	77.08 ± 27.72	79.86 ± 26.46	86.54 ± 18.87	87.18 ± 17.20	85.90 ± 16.12	83.33 ± 17.00
Symptom scales (higher scores = more symptoms)									0.667	0.123	0.190	0.425	0.659	0.140
Fatigue	37.50 ± 20.67	36.11 ± 18.89	34.72 ± 20.54	33.80 ± 21.85	35.47 ± 18.06	34.19 ± 17.76	38.03 ± 17.26	35.47 ± 19.88
Nausea and vomiting	6.94 ± 12.93	5.56 ± 11.70	4.17 ± 10.13	4.17 ± 10.13	5.13 ± 11.32	5.13 ± 11.32	5.13 ± 11.32	5.77 ± 11.49
Pain	32.64 ± 24.81	31.94 ± 23.01	29.86 ± 24.07	25.69 ± 24.56	25.64 ± 20.13	24.36 ± 19.57	26.92 ± 18.90	27.56 ± 18.82
Dyspnea	31.94 ± 25.02	29.17 ± 20.41	23.61 ± 20.80	23.61 ± 20.80	26.92 ± 24.98	23.08 ± 20.59	24.36 ± 22.23	25.64 ± 21.72
Insomnia	29.17 ± 22.66	27.78 ± 27.22	26.39 ± 25.97	20.83 ± 21.56	34.62 ± 30.52	33.33 ± 31.27	43.59 ± 32.34	42.31 ± 34.71
Appetite loss	25.00 ± 28.23	22.22 ± 28.94	18.06 ± 25.97	15.28 ± 25.97	12.82 ± 19.04	14.10 ± 21.44	15.38 ± 23.53	12.82 ± 23.24
Constipation	15.28 ± 21.93	20.83 ± 23.70	19.44 ± 27.66	15.28 ± 24.04	21.79 ± 22.98	17.95 ± 19.39	17.95 ± 21.56	14.10 ± 19.26
Diarrhea	15.28 ± 19.61	8.33 ± 14.74	9.72 ± 18.33	12.50 ± 19.19	12.82 ± 19.04	14.10 ± 19.26	14.10 ± 21.44	11.54 ± 18.72
Financial difficulties	26.39 ± 31.05	26.39 ± 32.57	20.83 ± 33.78	19.44 ± 32.48	12.82 ± 19.04	14.10 ± 19.26	15.38 ± 16.95	17.95 ± 19.39

EORTC QLQ-H&N35														
Symptom scales (higher scores = more symptoms)									0.804	0.317	0.572	0.003*	0.804	0.317
Pain	12.68 ± 13.02	12.68 ± 13.73	14.13 ± 16.94	10.87 ± 12.67	17.75 ± 24.27	19.20 ± 17.84	19.20 ± 16.56	18.48 ± 16.47
Swallowing	34.03 ± 25.05	31.94 ± 26.66	34.38 ± 25.81	34.72 ± 24.90	29.32 ± 18.69	28.70 ± 17.95	28.09 ± 17.01	26.23 ± 16.13
Sense problems	22.22 ± 18.82	21.53 ± 17.36^b^	18.75 ± 17.93^b^	16.67 ± 14.74^b^	33.95 ± 28.67	35.80 ± 26.03	34.57 ± 26.52	33.95 ± 25.94
Speech problems	29.17 ± 24.91	28.24 ± 25.38^b^	27.78 ± 25.80^b^	23.61 ± 23.47	18.52 ± 22.85	14.81 ± 19.49	13.99 ± 19.14	12.76 ± 18.92
Trouble with social eating	39.24 ± 32.37	36.81 ± 31.94	35.07 ± 33.24	33.33 ± 32.88	27.78 ± 29.87	25.93 ± 25.04	27.47 ± 24.11	25.62 ± 24.88
Trouble with social contact	21.45 ± 24.37	19.13 ± 21.40	18.84 ± 23.32	16.81 ± 22.64	12.46 ± 20.03	13.33 ± 19.07	9.28 ± 18.83	9.57 ± 19.47
Less sexuality	39.86 ± 33.99	36.23 ± 30.84	34.06 ± 30.76	39.86 ± 34.36	35.51 ± 29.86	42.03 ± 30.51	46.38 ± 34.07	46.38 ± 34.07
Teeth	44.44 ± 27.22	38.89 ± 23.40	37.50 ± 28.34	38.89 ± 27.22	39.51 ± 30.71	34.57 ± 31.33	38.27 ± 32.95	43.21 ± 33.10
Opening mouth	36.11 ± 30.95	30.56 ± 30.95	33.33 ± 31.08	31.94 ± 30.26	28.40 ± 30.25	32.10 ± 32.66	34.57 ± 32.66	32.10 ± 29.93
Dry mouth	65.28 ± 25.02	56.94 ± 23.01	55.56 ± 23.40	55.56 ± 23.40	62.96 ± 29.72	61.73 ± 28.80	62.96 ± 28.24	56.79 ± 30.40
Sticky saliva	44.44 ± 32.10	38.89 ± 30.56	38.89 ± 30.56	38.89 ± 30.56	48.15 ± 36.20	50.62 ± 35.05	50.62 ± 28.30	50.62 ± 31.17
Coughing	25.00 ± 22.52	23.61 ± 23.01	25.00 ± 24.57	25.00 ± 24.57	29.63 ± 31.12	19.75 ± 24.91	17.28 ± 16.97	18.52 ± 19.25
Felt ill	33.33 ± 20.60	28.99 ± 25.23	24.64 ± 27.00	24.64 ± 25.06	30.43 ± 28.27	31.88 ± 30.94	36.23 ± 28.27	33.33 ± 22.47

Pre: pretest (baseline); mid: midintervention test (3 months); post: posttest (6 months); FU: follow-up test (12 months).

Mean ± SD presented the continuous variables.

The effect sizes are in partial eta-squared.

Group-by-time interaction, group and time effects.

*Significant difference at *P* < 0.05.

Within group (alpha adjusted to 0.008 due to six paired comparisons).

^a^Significant difference at *P* < 0.008 compared with the pretest values.

Between groups.

^b^Difference significant at *P* < 0.05 compared with the control group.
